# Early impacts of the PEPFAR stop‐work order: a rapid assessment

**DOI:** 10.1002/jia2.26423

**Published:** 2025-02-18

**Authors:** Elise Lankiewicz, Alana Sharp, Patrick Drake, Jennifer Sherwood, Brian Macharia, Michael Ighodaro, Brian Honermann, Asia Russell

**Affiliations:** ^1^ Andelson Office of Public Policy, amfAR Washington DC USA; ^2^ Data, Et cetera Washington DC USA; ^3^ Health Global Access Project (GAP) Nairobi Kenya; ^4^ Global Black Gay Men Connect Washington DC USA; ^5^ Health Global Access Project (GAP) Washington DC USA

On 20 January, the Trump Administration issued an Executive Order freezing all foreign assistance funds for 90 days, to assess their alignment with the Administration's foreign policy priorities [1]. The freeze included funds disbursed under the U.S. President's Emergency Plan for AIDS Relief (PEPFAR), a historically bipartisan programme that has provided lifesaving HIV services since 2003. PEPFAR programmes are implemented primarily by the U.S. Centers for Disease Control and Prevention (CDC) and the United States Agency for International Development (USAID) and delivered by more than 450 prime implementing partners and around 850 sub‐recipients in 55 countries. Following this order, all U.S. embassies were ordered to immediately suspend all foreign assistance, with only limited exceptions for emergency food assistance and military financing for Egypt and Israel, as well as some administrative costs [2]. This sudden cessation of services, including HIV treatment, put millions of people at risk. Estimates predict that each day of the freeze about 220,000 people, including over 7000 children, will be unable to access their needed treatment [3].

On 1 February, a waiver was granted to PEPFAR, allowing the resumption of life‐saving humanitarian assistance during the review period [4]. The exemption was limited to diagnostics, treatment, management of opportunistic infections, supply chain support and certain human resources [4]. All HIV prevention activities, including the provision of pre‐exposure prophylaxis, were excluded from the waiver, except for those aimed at preventing mother‐to‐child transmission [4]. Further details on activities covered by the waiver were outlined in a Global Health Security and Diplomacy memo on 6 February [5]. However, the process for resuming services under the waiver still requires notification from a contracting or agreement officer and approval of a modified workplan and budget. As of 21 January, the CDC has been under orders not to communicate with external partners, and as of 8 February, almost all USAID staff were put on administrative leave [6, 7].

Measuring and urgently addressing the disruption to PEPFAR‐supported programmes is critical to save lives and mitigate the impact of the funding freeze, particularly given PEPFAR's own data systems have been shut down, eliminating their ability to track impacts on services [3, 8]. We surveyed PEPFAR funding recipients the week immediately following the funding freeze and stop‐work order (24 January−28 January 2025) using a web‐based survey tool available in English, French, Spanish, Portuguese, Russian and Thai. Respondents were recruited via listservs and WhatsApp groups relevant to the global HIV response. All individuals employed by a PEPFAR prime implementing partner or sub‐recipients were eligible to participate. Respondents were asked about the impact of the stop work order on service delivery, staffing and the ability to continue operating during the funding freeze. Data were collected anonymously, although 75% of respondents agreed to optionally report their organization name and countries of operation to facilitate deduplication.

After deduplication of organizations, 153 eligible respondents from 27 countries were included in this analysis. The majority of respondents were locally based (67%) and international (19%) nongovernmental organizations, while a smaller proportion represented local and international faith‐based organizations (4%), host country government agencies (3%) and other organizational types. Most respondents represented prime implementing partners (59%), with a majority of responses (61%) coming from Eastern and Southern Africa. A second smaller round of data collection was conducted from 1 February – 9 February focusing specifically on the waiver and included both survey data and qualitative data from PEPFAR partners. These data came from 65 respondents.

The findings from this survey reveal that the funding freeze has already led to significant disruptions in PEPFAR recipients’ ability to deliver HIV services. In the week immediately following the funding freeze, respondents reported:

- **Services**: PEPFAR partners reported widespread disruption of HIV services, defined as either cancelling or reducing activities that were previously offered. Overall, 71% of respondents reported having completely cancelled at least one category of activities. The services most frequently either cancelled or reduced were loss to follow‐up services and re‐engagement in care (94%), gender‐based violence services (92%), monitoring and data collection (91%), HIV testing (91%) and HIV treatment (91%) (Figure 1). The ability of implementers to maintain treatment programmes during the freeze was severely limited, with 86% of respondents reporting that their clients would lose access to HIV treatment services within the next month unless the freeze is lifted.
- **Staff layoffs and clinic closures**: Data show that the freeze has significantly impacted clinic staffing and operations. Over 60% of respondents reported that their organizations had already laid off staff. The most common layoffs occurred among community‐based staff, reported by 58% of respondents, while 47% had terminated organizational staff and 35% had terminated clinical staff. Nearly one‐third of organizations reported closing healthcare clinics across 16 countries, while 20% reduced their clinic hours of operation.
- **Financial stability**: The majority of PEPFAR implementers (76%) described the impact of the funding freeze on their organizational financial stability as “severe.” This was driven by a significant organizational reliance on PEPFAR funding, with nearly two‐thirds of respondents indicating that 75% or more of their funding is from PEPFAR.
- **Organizational closures**: Nearly one‐third of surveyed respondents said that their organizations had already completely closed down, either temporarily or permanently. Others indicated that they faced imminent closure unless funding was restored, with 7% needing funding restored within 1 week in order to avoid closure, and another 12% within 1 month. Only 14% of organizations reported that they could continue operating for more than 1 month without PEPFAR support.
- **The waiver**: As of 9 February, the waiver has not reached PEFPAR partners and most services remained paused. Of the 65 PEPFAR partners surveyed, less than 10% had restarted providing any services, and only 45% had received any official communications about the waiver. Qualitative data described multiple bureaucratic hurdles to services restarting. This included a slow process for approving modified work plans and budgets, being locked out of payroll or data systems, a fear of using funds without sufficient approval and ultimately needing to be repay those funds, and being unable to deliver approved services due to a financial reliance on activities frozen by the waiver.

**Figure 1:**
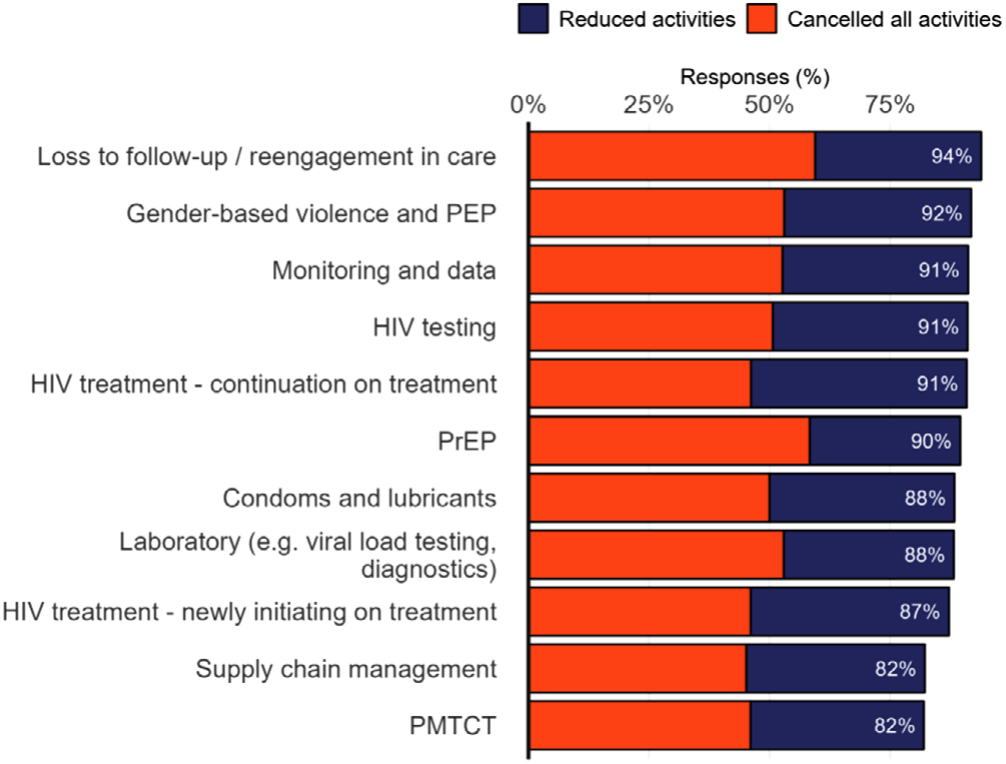
Disruptions of HIV services by activity, among organizations that provide these services. Abbreviations: PEP, post‐exposure prophylaxis; PrEP, pre‐exposure prophylaxis; PMTCT, prevention of mother‐to‐child transmission.

These findings indicate that the stop‐work order has had devastating consequences for PEPFAR beneficiaries. Although the waiver lifts portions of the funding freeze, these data highlight the inability of PEFPAR's global implementer network to absorb a complete financial shutdown without causing major disruption to public health services. Widespread reports of staff layoffs and clinic closures suggest that even if the waiver is clearly and immediately communicated, the resumption of service delivery will not be easily implemented.

The lack of diversified funding sources for PEPFAR‐implementing organizations observed in these data suggests that even short pauses in funding will be unsurvivable for many. Organizations that deliver HIV services not covered by the waiver, such as those primarily implementing prevention services, leading demand creation or providing peer support services, are especially unlikely to survive the 90‐day freeze. These services are the backbone of the overall functional health systems that have enabled countries to reach their 95‐95‐95 targets (95% of people living with HIV diagnosed, 95% of those diagnosed are on antiretroviral therapy (ART), 95% of those on ART are virally suppressed) and cannot be rebuilt overnight when the funding freeze is lifted if these organizations close. Without a strong network of HIV providers, the national and global HIV response will fall back from these hard‐fought gains and create the environment for HIV to re‐surge.

The funding freeze has already had immense consequences on the HIV response; some of them irreversible. UNAIDS estimates that since the funding freeze went into effect, more than 2000 people have acquired HIV who would otherwise have received PEPFAR‐supported prevention services [9]. If PEPFAR were to be eliminated during the 90‐day review, the world would be faced with a full resurgence of the HIV pandemic by 2029, with an estimated 6.3 million AIDS‐related deaths and 3.4 million AIDS orphans within 4 years [9]. While the waiver is an important step to re‐start HIV treatment and other critical components of the PEPFAR programme, it has not been clearly communicated or implemented and excludes entire tranches of prevention, care and support activities which are vital to the HIV response. Rapid waiver‐related workplan and budget approvals are urgent and essential to mitigating additional disruption. However, the waiver alone will be insufficient to protect the HIV response. Lifting the funding freeze immediately is imperative to prevent further backsliding of progress towards ending the global HIV epidemic—progress that would not have been possible without PEPFAR's 27 years of leadership.

## COMPETING INTERESTS

The authors have no competing interests to declare.

## AUTHORS’ CONTRIBUTIONS

AS, PD and EL conceptualized the survey and report. PD led data analysis. AS, EL and JS wrote the initial draft. BH, AR, BM and MI reviewed and contributed to revisions.

## FUNDING

No external funding was obtained for this work.

## Data Availability

The data that support the findings of this study are available on request from the corresponding author. The data are not publicly available due to privacy or ethical restrictions.

## References

[1] The White House . Reevaluating and realigning United States foreign aid [Internet]. 2025. [cited 2025 Feb 3]. Available from: https://www.whitehouse.gov/presidential‐actions/2025/01/reevaluating‐and‐realigning‐united‐states‐foreign‐aid/

[2] Hansler J . US freezes almost all foreign aid. CNN; 2025. [cited 2025 Feb 3]; Available from: https://www.cnn.com/2025/01/24/politics/us‐freezes‐foreign‐aid/index.html

[3] Andelson Office of Public Policy . Impact of stop work orders for PEPFAR programs [Internet]. amfAR, The Foundation for AIDS Research; 2025. [cited 2025 Feb 3]. Available from: https://www.amfar.org/wp‐content/uploads/2025/01/Impact‐of‐Stop‐Work‐Orders‐for‐PEPFAR‐Programs‐2.pdf

[4] Kates J . The status of President Trump's pause of foreign aid and implications for PEPFAR and other global health programs [Internet]. 2025. [cited 2025 Feb 3]. Available from: https://www.kff.org/policy‐watch/the‐status‐of‐president‐trumps‐pause‐of‐foreign‐aid‐and‐implications‐for‐pepfar‐and‐other‐global‐health‐programs/

[5] Global Health Security and Diplomacy . HIV care & treatment and prevention of mother to child transmission activities approved under PEPFAR limited waiver [Internet]. United States Department of State; 2025. [cited 2025 Feb 7]. Available from: https://www.unaids.org/sites/default/files/media_asset/GHSD_PEPFAR‐Limited‐Waiver‐Approved‐Activities.pdf

[6] Knickmeyer E , Lee M . Trump's administration is pulling almost all USAID workers off the job worldwide. Associated Press; 2025. [cited 2025 Feb 6]; Available from: https://apnews.com/article/trump‐usaid‐layoffs‐7e0a159d8a419c4c9388ab02e8259f23

[7] Emanuel G , Simmons‐Duffin S . Federal health agencies told to halt all external communications. National Public Radio; 2025. [cited 2025 Feb 6]; Available from: https://www.npr.org/sections/shots‐health‐news/2025/01/22/nx‐s1‐5270866/hhs‐cdc‐health‐communications‐trump

[8] Mandavilli A . Trump administration halts H.I.V. drug distribution in poor countries. The New York Times; 2025. [cited 2025 Feb 6]; Available from: https://www.nytimes.com/2025/01/27/health/pepfar‐trump‐freeze.html

[9] Joint United Nations Programme on HIV/AIDS (UNAIDS) . Impact of recent U.S. shifts on the global HIV response [Internet]. 2025. [cited 2025 Feb 6]; Available from: https://www.unaids.org/en/topic/PEPFAR_impact

